# Retraction: Diagnostic and Prognostic Utility of High-Sensitivity Troponin T (hs-TNT) and HEART Score in Risk Stratification of Acute Chest Pain in the Emergency Department

**DOI:** 10.7759/cureus.r215

**Published:** 2026-01-06

**Authors:** Muhammad Tayyab, Muhammad Farooq, Muhammad Subhan Javed Butt, Tamilarasu Gunasekaran, Aisha Bashir, Murad Ali, Muhammad Arsalan, Muhammad Afnan, Muhammad Irfan Ullah

**Affiliations:** 1 Trauma and Orthopaedics, Hayatabad Medical Complex Peshawar, Peshawar, PAK; 2 Internal Medicine, Nishtar Medical University, Nishtar-II Hospital, Multan, PAK; 3 Emergency Medicine, Royal Lancaster Infirmary, Lancaster, GBR; 4 Emergency Medicine, Shifa International Hospital Islamabad, Islamabad, PAK; 5 Emergency Medicine, Kampar Hospital, Perak, MYS; 6 Internal Medicine, Omar Hospital and Cardiac Centre, Lahore, PAK; 7 Internal Medicine, Bacha Khan Medical College, Medical Teaching Institution (MTI) Mardan Medical Complex, Mardan, PAK; 8 Internal Medicine, Pakistan Institute of Medical Sciences (PIMS) Hospital, Peshawar, PAK; 9 Internal Medicine, Divisional Headquarter Hospital, Kohat, PAK; 10 Internal Medicine, Saidu Medical College, Swat, PAK

This article has been retracted by the Editor-in-Chief due to serious concerns regarding the reliability and integrity of the reported data and analyses. The article contains internally inconsistent primary outcome counts, with substantially different 30-day and 45-day MACE numbers reported in the abstract compared with the results, tables, and figures, rendering the core findings unreliable. Additionally, the statistical analyses are fundamentally flawed by circular modeling that double-counts troponin through its combination with the HEART score, invalidating the reported predictive performance and conclusions. The authors agree with the decision to retract.

